# A Giant Compressive Mesenteric Lipoblastoma Initially Suspected to Be Abdominal Malignancy: A Report of a Rare Case in a Nine-Month-Old Infant

**DOI:** 10.7759/cureus.33799

**Published:** 2023-01-15

**Authors:** Rita Cempaka S, Lina Choridah, Vincent Lau, Andrew Nobiantoro Gunawan, Vincent Laiman, Bambang Ardianto, Didik S Heriyanto

**Affiliations:** 1 Anatomical Pathology, Universitas Gadjah Mada/Dr. Sardjito General Hospital, Yogyakarta, IDN; 2 Radiology, Universitas Gadjah Mada/Dr. Sardjito General Hospital, Yogyakarta, IDN; 3 Child Health, Universitas Gadjah Mada/Dr. Sardjito General Hospital, Yogyakarta, IDN

**Keywords:** malignancy, mesenteric, compressive, paediatric oncology, soft tissue pathology, lipoblastoma

## Abstract

Lipoblastoma is a rare benign soft tissue neoplasm rising from embryonic white adipose tissue known as lipoblast that keeps proliferating during the postnatal period. Although lipoblastomas are benign, they often grow rapidly. Most lipoblastomas are asymptomatic at presentation; they can present as a growing painless palpable mass and progressive symptoms of various organ compression depending on localization. A giant mesenteric lipoblastoma is a rare case with only a few cases reported. An infant with large intraabdominal masses may present preoperative diagnostic difficulties. Differential diagnoses are broad and may include sarcomas, germ-cell tumors, lipomas, lymphomas, hepatoblastomas, Wilm’s tumors, and neuroblastomas. Thorough clinical, radiological, and pathological investigations are ultimately required to obtain a definitive diagnosis. Regardless of location, the treatment of choice for lipoblastoma is complete surgical resection. All patients should be followed up for a minimum of five years

We report a rare case of a giant compressive mesenteric lipoblastoma that was initially suspected as abdominal malignancy in a nine-month-old infant. As physicians, we must always consider the underlying cause as well as the malignant or benign nature of a growing mass to treat the patient appropriately.

## Introduction

Lipoblastoma is a rare benign soft tissue neoplasm originating from embryonic white adipose tissue known as lipoblast that continues to proliferate during the postnatal period [1-5]. There are two subtypes of this tumor: (1) lipoblastoma which originates in the superficial layer as a localized well-circumscribed lesion, and (2) lipoblastomatosis which originates in deep soft tissue and has an infiltrative pattern rendering a more severe characteristic and higher recurrence [1,3,6]. Although this distinction may not be clinically important, because both can recur [1,2], it is crucial to know that these are two separate entities histopathologically. Lipoblastoma's important differential diagnosis is myxoid or well-differentiated liposarcoma [3,5]. Lipoma, fibrolipoma, hibernoma, lipofibromatosis, and fibrous hamartoma are other histologically similar lesions but they lack lipoblasts [3].

Lipoblastoma primarily occurs in infancy and early childhood, with approximately 90% of cases occurring before the age of three and having a slight male predominance. The extremities and trunk are the most prevalent sites [1,7,8]. Other sites including the head and neck, retroperitoneum, pelvis, and abdomen are less prevalent [7,8]. Lipoblastoma has also been reported in the lung, heart, colon, and parotid gland [1]. Lipoblastoma, although benign with no risk of metastasis [1], may grow rapidly, compressing adjacent structures and if large enough, may cause symptoms due to the mass effect [5]. Complete resection with free margin is the only known definitive treatment, additional considerations might be required in specific sites such as extremities [5,9]. 

We report a case of a giant compressive benign mesenteric lipoblastoma in a nine-month-old infant presenting with a painless growing abdominal mass with no notable symptoms.

## Case presentation

A nine-month-old infant was referred to our pediatric surgery clinic for evaluation of a distended abdomen. He presented with an enlarging mass on the left side of his abdomen with an abnormal ultrasonography (USG) examination provided by the referring pediatrician. He was born pervaginally with no complications perinatally. There were no obvious congenital anomalies at birth, nor was there a family history of sickness. At the age of eight months, the infant was first brought to a healthcare facility by his parents with the chief complaint of an enlarging mass on the left side of his abdomen. His parents had noticed the growing mass two months ago. The mass was said to be growing rapidly. At the first presentation, the mass measured about the size of a tennis ball (~6 cm ✕ 6 cm) at the left hypochondriac region. There were no notable symptoms other than a delay in his gross-motor developmental milestones (he was not able to sit by himself yet). The USG examination revealed there was a left suprarenal inhomogeneous mass with suspicion of neuroblastoma. The patient was subsequently referred to the pediatric surgery department at our hospital for further investigation of abdominal malignancy.

**Figure 1:**
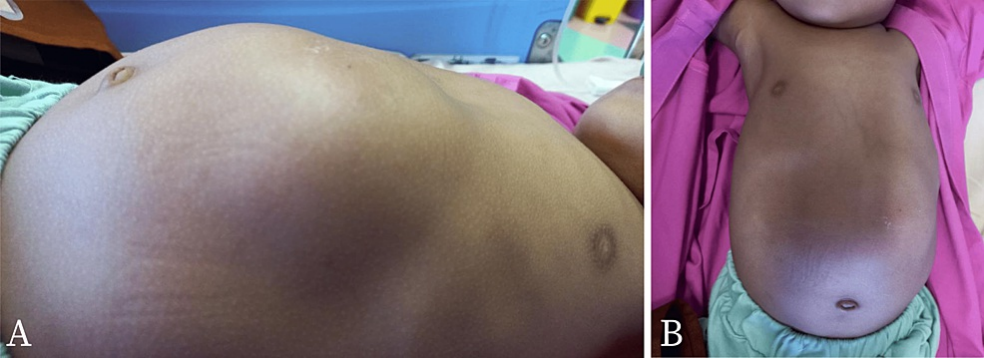
Physical examination of the patient’s abdomen Patient's distended abdomen with a protruding mass measuring about 10 cm x 10 cm (A) with superficially visible engorged veins (B)

**Figure 2:**
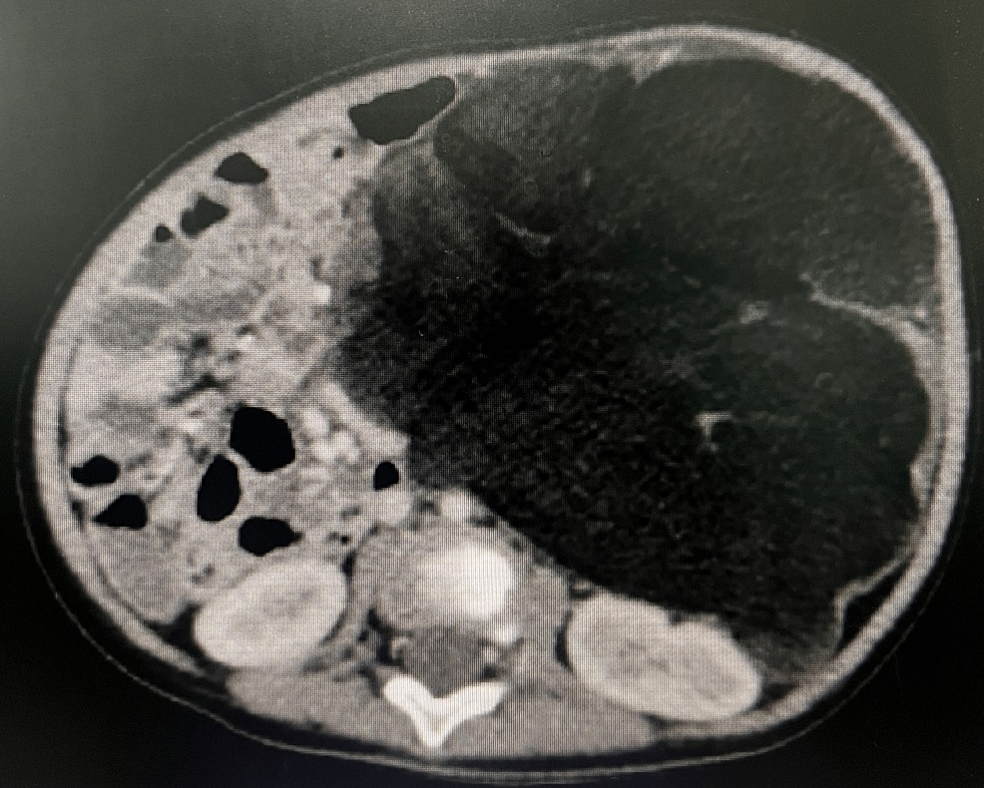
Abdominal CT scan of the patient Seen is a large intraperitoneal multilobulated mass with a fat component in the anterior of the left kidney

The patient came to our hospital one month after the first encounter with the healthcare provider. The patient’s abdomen was distended (Figure 1 A) with a protruding mass measuring about 10 cm x 10 cm with engorged veins visible superficially (Figure 1 B). From palpation, there was a lobulated mass with a distinct margin, it was soft, mobile, and not tender. A more advanced modality was then used to determine the origin and the advancement of the suspected malignancy. Tumor marker exploration of germ cell tumors (GCTs) such as alpha-fetoprotein (AFP), beta-human chorionic gonadotropin (hCG), cancer antigen 19-9 (CA 19-9), and lactate dehydrogenase (LDH) showed normal values. Contrast-enhanced CT of the thorax and abdomen was performed. Radiologic examination revealed the finding of a large intraperitoneal multilobulated mass with a fat component in the anterior of the left kidney measuring anteroposterior (AP) 11.9 cm ✕ latero-lateral (LL) 11.3 ✕ craniocaudal (CC) 12.5 cm that is enlarging to the contralateral side, compressing the left kidney to the posterior, spleen to craniolateral, pancreas and bowel system to the right side (Figure 2). This finding suggested mesenterium lipoblastoma. 

**Figure 3:**
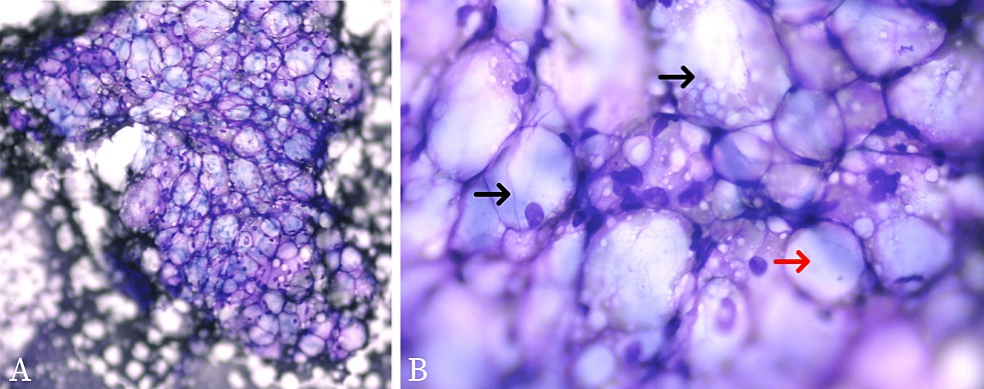
Cytopathology photomicrograph from fine needle aspiration biopsy A: Adipocytes and lipoblasts on low magnification, 40x; B: Mature adipocytes (red arrow) and lipoblasts, both with uni-vacuolated and multi-vacuolated features (black arrow), 100x

**Figure 4:**
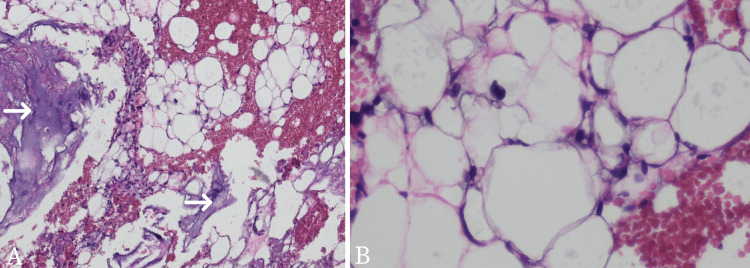
Cytopathology photomicrograph examination from the cell block A: Immature lipid cells known as lipoblast arranged as lobules with a myxoid background (white arrow), H&E, 40x; B: Large lipid cells with clear cytoplasm with their nucleus located at the edge of the cell, H&E, 100x H&E: Hematoxylin and eosin

**Figure 5:**
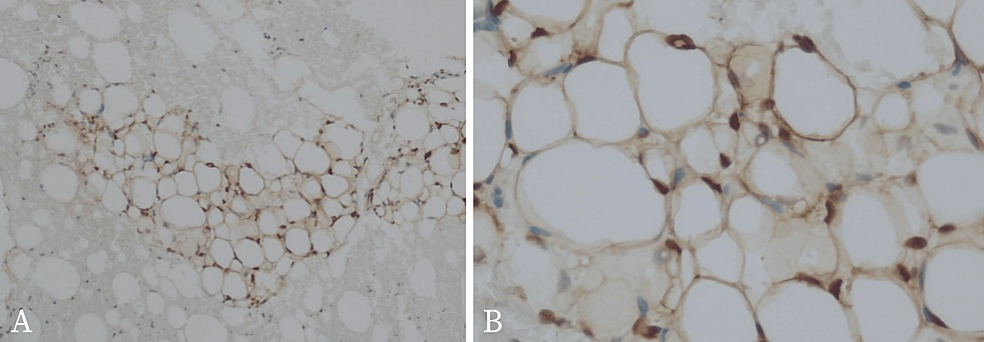
Immunohistochemistry (IHC) analysis with anti-S100 antibody The lipoblast nuclei are strongly positive for anti-S100 antibody staining

Based on the physical and radiologic examination, the doctor ordered an USG-guided fine needle aspiration biopsy (FNAB) to be done at the targeted mass. Cytopathology showed mature adipocytes and lipoblasts, both uni-vacuolated and multi-vacuolated lipoblasts (Figure 3). Cytopathology examination from the cell block (Figure 4) revealed immature lipid cells known as lipoblast that arranged as lobules with a myxoid background. Large lipid cells with clear cytoplasm and their nucleus located in the center and the edge of the cell. There were also some spindle cells but no malignant cells were found. In immunohistochemistry (IHC) analysis (Figure 5), the lipoblast nuclei are strongly positive for anti-S100 antibody staining. These findings are consistent with the previous results and support the diagnosis of a benign mass, a lipoblastoma.

**Figure 6:**
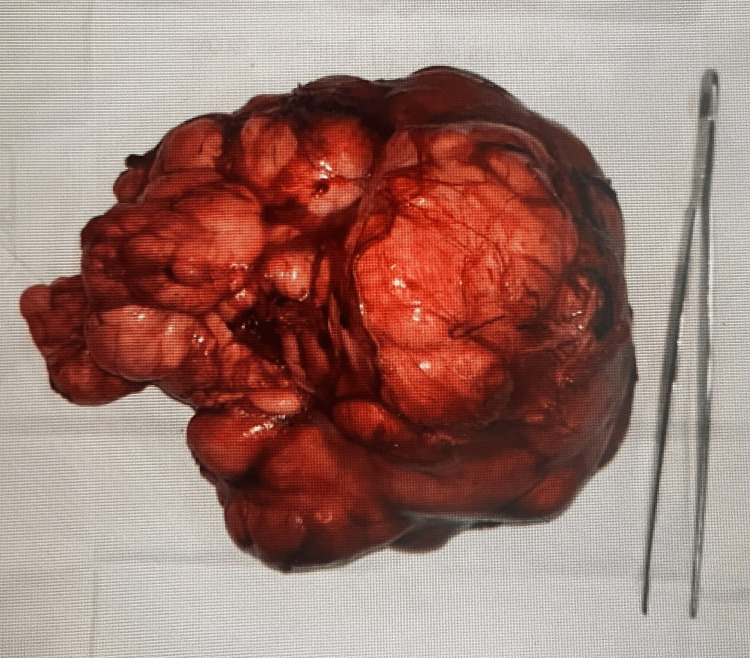
Resected mass The mass was soft and measured 16.5 cm x 11.2 cm x 6.5 cm grossly

**Figure 7:**
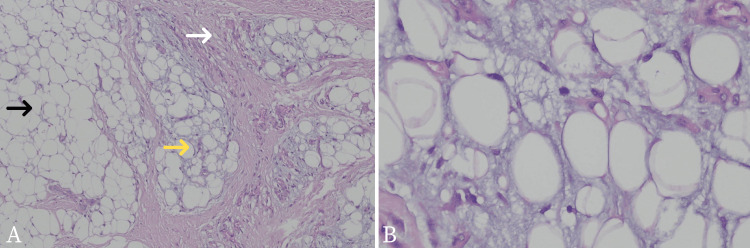
Histopathology examination from the resected mass A: Lipid tissues arranged in lobules separated by fibrous tissue (white arrow) with a scattered myxoid background. Some fields (black arrow) consist of mature adipocytes, and the other contains lipoblasts (yellow arrow); B: Higher magnification of lipoblasts which shows no presence of malignant cells

The pediatric surgeon decided to do a laparotomy and radical resection on the suspected benign mass (Figure 6). The procedure was carried out two months after the first encounter with the healthcare provider. It was carried out with minimum bleeding and there were no complications perioperatively. The sample was sent to the anatomical pathology department for a histopathology examination. The sample was soft and measured 16.5 cm x 11.2 cm x 6.5 cm. Microscopically, there were lipid tissues arranged in lobules separated by fibrous tissue with some fields showing myxoid background (Figure 7). There were lipoblasts but no malignant cells were found. These results were consistent with lipoblastoma. Due to insufficient infrastructure and funds, the pleomorphic adenoma gene 1 (PLAG1) gene examination was not performed at our institution. The patient was then discharged from the hospital four days after the surgery. Follow-up was done one week after the surgery, and the patient’s recovery progress was uneventful. 

## Discussion

Adipose and myxoid tumors in children are uncommon, and the majority are benign. In the first two decades of life, less than 10% of soft tissue lesions are tumors originating from adipose tissue [3]. Lipoblastoma is mostly diagnosed exclusively during infancy, childhood, and adolescence. A review included 263 cases (13 case series) of lipoblastoma with a median age at presentation of 22.6 months [10], and a 1.7:1 male-to-female ratio, which indicates a slight male predominance [8,10]. Abdominal lipoblastomas are rare; around 7% of all lipoblastomas and the majority of them develop retroperitoneally [6,11,12]. Lipoblastoma occurs in 80% of cases, whereas lipoblastomatosis occurs in 20% of cases [13]. Lipoblastoma and lipoblastomatosis are benign and do not have a risk to metastasize or undergo malignant transformation [1,3,4, 6,13], the natural history of an unresected lipoblastoma may be maturing into a lipoma [4,6,11]. The recurrence rate reported ranges from 13% to 46% in seven case series [8]. In the 214 cases studied, the average recurrence rate was 17% [10]. 

Although lipoblastomas are benign, they often grow rapidly. Most lipoblastomas are asymptomatic at presentation and can present as a growing painless palpable mass with progressive symptoms of various organ compression depending on localization [5,6,14]. Patients with pleural, mediastinal, pulmonary, and lower neck lipoblastomas have been reported to experience airway obstruction and respiratory difficulties [6]. Patients with mesenteric or retroperitoneal lipoblastomas experience gastrointestinal symptoms, such as emesis, diarrhea, anorexia, and abdominal pain [6]. These symptoms were not found in our patient. Developmental delays with motor incoordination and/or a nonspecific developmental delay have been reported in children with lipoblastoma [8]. As in our case, a delay in gross motoric development was likely to be caused by the abdominal mass hindering the movement of the child.

A giant mesenteric lipoblastoma is a rare case with only a few cases reported [15-17]. There were 22 cases reported from 1973 to 2020 [16]. The tumor sizes ranged from 12 cm to 23 cm in diameter [16]. Others, have also reviewed retroperitoneal lipoblastomas and found 19 reported cases [18]. The tumors varied in size from 8 cm to 25 cm (median 15.5 cm). In 10 different studies of patients with lipoblastoma in varying sites, the retroperitoneal site was consistently the greatest in size compared with lipoblastoma at other sites [18]. Our case is one of the largest mesenteric lipoblastoma reported to date. An infant with large intraabdominal masses may present preoperative diagnostic difficulties. Differential diagnoses are broad and may include sarcomas, germ-cell tumors, lipomas, lymphomas, hepatoblastomas, Wilm’s tumors, and neuroblastomas [18]. Large masses are usually associated with more aggressive tumors and tend to be malignant tumors. Investigation of the mass may include tumor biomarkers, USG evaluation of soft tissue tumors, CT scans for mass characterization and lymphadenopathy detection, and MRI for particular masses and anatomic delineation [18,19].

In our case, the pediatrician first diagnosed the patient with neuroblastoma from the clinical and the USG findings. Neuroblastoma is the most common intra-abdominal and extracranial solid tumor found in infants [20]. With our patient, a contrast-enhanced CT scan was able to characterize the tumor. For both preoperative diagnosis and follow-up, MRI remains the imaging method of choice [18-21]. An MRI provides the highest sensitivity for the pathology of tumors since the increased vascularity and cellularity in lipoblastomas, compared to lipomas, shows a lower intensity on T1-weighted images [5,18-21]. Also in T1-weighted images, the lipoblast fat signals are usually less intense than the signal of mature fat cells (adipocytes) in lipoma [19]. If feasible, ultrasound-guided FNAB for lipoblastoma evaluation are well correlated with the histopathological findings and may aid in the preoperative diagnosis or follow-up of recurrences [18,19,22]. The lipoblast demonstrates positive for S100, CD34, and C56 and is often positive with desmin [1, 3]. In our case, cytological analysis and the positive staining with IHC S100, which were performed preoperatively, were able to diagnose the suspected mass. However, thorough clinical and pathological investigations are ultimately required to obtain a definitive diagnosis.

Histologically, lipoblastoma characteristically demonstrates lobulated architecture formed of sheets of adipocytes with variable maturation (primitive mesenchymal cells or spindle cells to multivacuolated or small signet ring lipoblast and some mature adipocytes) separated by fibrovascular septa. Lipoblastoma may exhibit a zonal pattern of maturation with more immature myxoid cells at the periphery adjacent to fibrous septa, and mature adipocytes at the center of the lobule. The myxoid regions can occasionally show pooling of matrix similar to myxoid liposarcoma. However, mature areas may resemble lipoma [1,3]. Differential diagnoses of lipoblastoma may include pediatric lipoma, hibernoma, pediatric myxoid liposarcoma, and well-differentiated liposarcoma. Lipomas are lacking lipoblasts, whereas hibernomas are composed of brown fat cells with a central nucleus and abundant finely granular cytoplasm. Lipomas are also not common in extremities. Myxoid liposarcoma is exceedingly rare in children under the age of 10. Histologically, myxoid liposarcoma shows nuclear atypia and does not show a lobulated growth pattern. The zonal pattern of maturation is also different, whereas, in myxoid liposarcoma, maturation occurs towards the periphery [3,14]. Differentiating between lipoblastoma and myxoid liposarcoma is important for the treatment as the latter requires more aggressive treatment such as extensive resection with local radiotherapy [9,14].

If clinical and histologic examinations are not sufficient to differentiate between these two tumors, cytogenetic testing may be indicated [3,5,10,14,22]. Nearly 90% of lipoblastomas have chromosomal 8q13 rearrangements in the PLAG1 gene area or gains of chromosome 8 with PLAG1 amplification. Meanwhile, rearrangement t(12;16)(q13;p11) results in the fusion of fused in sarcoma (FUS)-DNA damage-inducible transcript 3 (DDIT3) genes, which is nearly found in 95% of cases of myxoid liposarcoma [3,5,14]. Immunohistochemistry might be useful for excluding diagnoses. Liposarcoma is positive for MDM2 and CDK4 while lipoblastomas are negative. Most of the myxoid liposarcoma and well-differentiated liposarcoma are positive for p16 while most of the lipoblastomas are negative [14]. Due to insufficient infrastructure and funds, the PLAG1 gene examination was not performed at our institution. Immunohistochemistry is a feasible choice to assist the diagnosis process, especially if molecular testing is not available in certain clinical settings. In our case, clinical and histological examinations were sufficient to diagnose lipoblastoma. 

Regardless of location, the treatment of choice for lipoblastoma is complete surgical resection [3,5,9,15]. Although, when dealing with large invasive lipoblastomas, a conservative “wait and see” method might be useful, especially if there are no life-threatening complications [5,23]. Avoiding extensive surgery that would lead to anatomical abnormalities in an infant might be an option, as there is one reported case of spontaneous resolution of an infant lipoblastomatosis in the left thigh at eight months of follow-up [23]. All patients should be followed up for a minimum of five years [5,9].

## Conclusions

In conclusion, we report a rare case of a giant compressive mesenteric lipoblastoma that was initially suspected as abdominal malignancy in a nine-month-old infant. In patients with a large distended abdomen that grows progressively, abdominal ultrasonography and CT scan aid in the initial diagnosis to adjust the preferred management. Meanwhile, histopathology and immunohistochemistry examinations emphasize the definitive diagnosis of these cases for the prognosis and treatment. As physicians, we must always consider the underlying cause as well as the malignant or benign nature of a growing mass in order to treat the patient appropriately.
